# Combining elemental and immunochemical analyses to characterize diagenetic alteration patterns in ancient skeletal remains

**DOI:** 10.1038/s41598-022-08979-3

**Published:** 2022-03-24

**Authors:** L. Gatti, Federico Lugli, Giorgia Sciutto, M. Zangheri, S. Prati, M. Mirasoli, S. Silvestrini, S. Benazzi, T. Tütken, K. Douka, C. Collina, F. Boschin, M. Romandini, P. Iacumin, M. Guardigli, A. Roda, R. Mazzeo

**Affiliations:** 1 Department of Chemistry, University of Bologna-Ravenna Campus, Via Guaccimanni, 42, 48121 Ravenna, Italy; 2 Department of Cultural Heritage, University of Bologna-Ravenna Campus, Via degli Ariani 1, 48121 Ravenna, Italy; 3 Department of Chemical and Geological Science, University of Modena and Reggio Emilia, 41125 Modena, Italy; 4 Department of Chemistry, “Giacomo Ciamician” Alma Mater Studiorum-University of Bologna, Via Selmi 2, 40126 Bologna, Italy; 5 Applied and Analytical Paleontology, Institute of Geosciences, Johannes Gutenberg University, 55128 Mainz, Germany; 6 Department of Archaeology, Max Planck Institute for the Science of Human History, 07745 Jena, Germany; 7 Research Laboratory for Archaeology and the History of Art, University of Oxford, Oxford, OX1 3QY UK; 8 Museo Civico Archeologico Biagio Greco, Mondragone, Caserta, Italy; 9 Department of Physical Science, Earth and Environment, U.R. Preistoria e Antropologia, University of Siena, Siena, Italy; 10 Department of Chemistry, Life Sciences and Environmental Sustainability, University of Parma, Parma, Italy; 11 INBB, National Institute of Biostructures and Biosystems, Rome, Italy

**Keywords:** Biogeochemistry, Chemistry, Bioanalytical chemistry, Imaging studies, Mass spectrometry, Archaeology, Palaeontology

## Abstract

Bones and teeth are biological archives, but their structure and composition are subjected to alteration overtime due to biological and chemical degradation *postmortem*, influenced by burial environment and conditions. Nevertheless, organic fraction preservation is mandatory for several archeometric analyses and applications. The mutual protection between biomineral and organic fractions in bones and teeth may lead to a limited diagenetic alteration, promoting a better conservation of the organic fraction. However, the correlation between elemental variations and the presence of organic materials (e.g., collagen) in the same specimen is still unclear. To fill this gap, chemiluminescent (CL) immunochemical imaging analysis has been applied for the first time for collagen localization. Then, Laser Ablation–Inductively Coupled Plasma–Mass Spectrometry (LA–ICP–MS) and CL imaging were combined to investigate the correlation between elemental (i.e., REE, U, Sr, Ba) and collagen distribution. Teeth and bones from various archeological contexts, chronological periods, and characterized by different collagen content were analyzed. Immunochemical analysis revealed a heterogeneous distribution of collagen, especially in highly degraded samples. Subsequently, LA–ICP–MS showed a correlation between the presence of uranium and rare earth elements and areas with low amount of collagen. The innovative integration between the two methods permitted to clarify the mutual relation between elemental variation and collagen preservation overtime, thus contributing to unravel the effects of diagenetic alteration in bones and teeth.

## Introduction

Bones and teeth are complex biomineralized tissues composed by a mineral fraction, mainly carbonate-substituted bioapatite, and an organic fraction, mainly collagen type I protein in bones and dentin^1–8^. After burial, bone structure and composition are subjected to diagenesis, which is the alteration induced by physical, biological, and chemical degradation^9–13^. Similarly, tooth dentin can be subjected to post-burial biological and chemical degradation^9,14–17^. Despite the lower resistance of bone and dentin to diagenesis, than the highly mineralized enamel, their analysis provides chemical and isotopic information on diet and provenance. Moreover, they also contain DNA and proteins, from which information on gender, taxonomy and evolution can be obtained for past reconstructions of human and faunal life histories. Yet, the preservation of the organic fraction is pivotal to obtain such information by archeometric investigations.

During diagenesis, the mineral fraction may be affected by the penetration of ground waters into the bone structure, inducing apatite recrystallization, changes in the elemental composition and adsorptions onto the surface of bioapatite crystals^18–20^. Some essential trace elements, abundant during life, decrease after burial while others, such as rare earth elements (REE) and uranium (U), are incorporated during diagenesis. Moreover, hydrolysis may induce the breakage of the peptide bonds, leading to the decomposition of proteinaceous materials^9,21–26^. These phenomena could result in a heterogeneous preservation of the organic fraction within the same specimen.

Among the investigated elements, U and REE are widely used as diagenetic proxies monitor the alteration degree of bones (and teeth) due to their remarkably low levels in fresh bones (< 1 µg/g). However, their effective correlation with the amount of preserved collagen has not yet been adequately assessed. During diagenesis, U and REE migrate from the soil to the bone tissues, mostly through groundwaters (i.e., pore fluid interaction). Particularly, being highly water soluble as uranyl (UO_2_^2+^), uranium is readily incorporated in fossil bones and represents a strong marker for post-depositional chemical alteration of the bone mineral portion^27,30–38^. Similarly, due to the high adsorption capacity of bone crystallites for REE^3+^^31^, their adsorption began immediately with the exposure of bone to pore water, yielding high REE content in fossil specimens (ΣREE up to several 1000 µg/g)^32^. Strontium (Sr) and barium (Ba) have been considered as specific biomarkers for trophic level and diet^33^. Indeed, they are widely used in dietary reconstruction, thus their physiological constraints are relatively well-known and provide a solid reference for studies on bone chemistry^33^. Yet, due to diagenetic processes that promote post-burial substitution of lattice Ca^2+^ by alkali elements, Sr^2+^ and Ba^2+^ are commonly enriched in fossil bones compared to fresh bones (~ 10 µg/g and ~ 200 µg/g respectively in human fresh bones)^24,27,34^.

The enrichment of abovementioned elements may be theoretically correlated to diagenetic and degradation effects on both inorganic and organic fraction of ancient bones. Indeed, bones and teeth that were affected only by a limited degree by diagenetic decay of the mineral component, usually show a better conservation of the organic fraction^26^. In contrast, when a strong diagenetic alteration of the mineral structure is observed, also the organic component shows a worse conservation status. This suggests a possible mutual protection between mineral and organic fractions, since the degradation of collagen expose the mineral fraction to water penetration (due to the increased porosity) promoting diagenetic processes and, simultaneously, the degradation of the mineral structure facilitates the water penetration with higher risk for collagen hydrolysis^9,26,28,35^.

According to this correlation, specific elements such as U and Sr, that are commonly used as diagenetic markers for the assessment of the state of conservation of the mineral components of bone and teeth, have also been used as markers for the evaluation of the organic fraction preservation in the same specimens^26,27,29,30,36–38^. Indeed, the low abundance of diagenetic elemental markers reflects a limited/no bioapatite alteration and thus should correspond to collagen preservation as both biomineral and the protein phase are tightly intergrown and mutually protect each other. In contrast, a high content of these elements indicates a stronger alteration and *postmortem* should also lead to a greater collagen loss. To date, the relationship between the variation of diagenetic markers and the presence of collagen in the same sample is still unclear and not adequately studied. Elemental and isotope analyses, based on the use of different analytical techniques such as synchrotron-based X-ray fluorescence^39,40^ and Laser Ablation-Inductively Coupled Plasma-Mass Spectrometry (LA–ICP–MS)^34,38,41–45^, have been applied to identify well-preserved areas (possible rich in collagen) to be sampled for studies concerning, e.g., dating, paleodiet reconstruction and taxonomic discrimination. Fourier Transform Infrared (FTIR) has also been applied to assess the degradation state of bone. In particular, amide-to-phosphate ratio and crystallinity index were used to evaluate the preservation of the organic fraction^12,46–50^. Near-infrared hyperspectral imaging (NIR-HSI) has been recently proposed as a prescreening method for collagen mapping in archeological bones in combination with a normalized difference image (NDI) data processing^51^.

Nevertheless, to date, no investigations have been conducted to evaluate the effective correlation between collagen distribution (irrefutably identified with high selective techniques) and elemental composition of the diagenetic markers in different areas of the same samples. Only recently, a study reported the parallel investigation of REE distribution and collagen on a single sample (a well-preserved dinosaur fibula). Collagen has been detected by combining enzyme-linked immunosorbent assay (ELISA) applied on protein extracts, and in situ immunofluorescence applied on bone fragments^26^. The results suggested the correlation between the low REE content with the presence of collagen.

The present study proposes a new analytical strategy for the characterization of diagenetic pathways in ancient bones and teeth, combining elemental and collagen distribution investigations obtained with LA–ICP–MS and chemiluminescence (CL) immunochemical microscopy imaging, respectively. In particular, CL immunochemical analysis was applied for the first time on archeological and fossil bones and tooth to sensitively localize collagen protein in complex biomineralized matrices, providing a new tool to screen for the presence of proteins, since the characterization of proteins in ancient skeletal remains may address research questions concerning diet and genetic relations.

Immunochemical methods exploit the high specificity of the antigen–antibody recognition reaction, ensuring the direct and specific detection of collagen^52–55^. These methods have been widely applied in bioanalysis and clinical chemistry and, over the past decade, have proven to be efficient methods for the selective identification of proteins in cultural heritage objects^52–61^.

The new approach proposed herein presents specific advantages that permits to overcome several limitations of previous research studies: (1) a higher number of samples from various archeological excavation sites and characterized by different chronological periods and collagen content have been considered; (2) the distribution of collagen was determined using CL immunochemical detection, which has proved to be more sensitive than immunofluorescence and highly selective in the specific identification of proteins in complex matrices^61^, also providing good spatial resolution and low background signal; (3) several diagenetic marker elements were quantified by LA–ICP–MS analysis, exploiting the combined use of single-spots and laser ablation imaging^62^. The latter permitted to obtain a distribution map of the element of interest, directly comparable with the CL collagen images, which further on could enable the identification of diagenetic marker elements (i.e., REEs and U) correlating with collagen preservation. The investigation of samples, from different period/age and excavation sites, allowed to assess the influence of different variables, which could affect diagenetic phenomena and thus collagen preservation, identifying elements that can be used as representative diagenesis extent markers. Age was the first variable considered in order to identify independent marker (i.e., from the Early Middle Ages to the Middle-Upper Paleolithic). In addition, samples from the same period but from different sites were considered to evaluate the impact of diagenesis on bones from different ecogeographical regions (i.e., Northern and Southern Italy).

Exploring the correlation between collagen and the distribution of diagenetic markers could be useful to deeper understand the phenomena promoting post-burial elemental uptake/leaching and biomolecule degradation/preservation. This, in turn, can help future works identifying and retrieving non-altered biogenic chemical signals from (fossil) bones.

## Results

**Figure 1:**
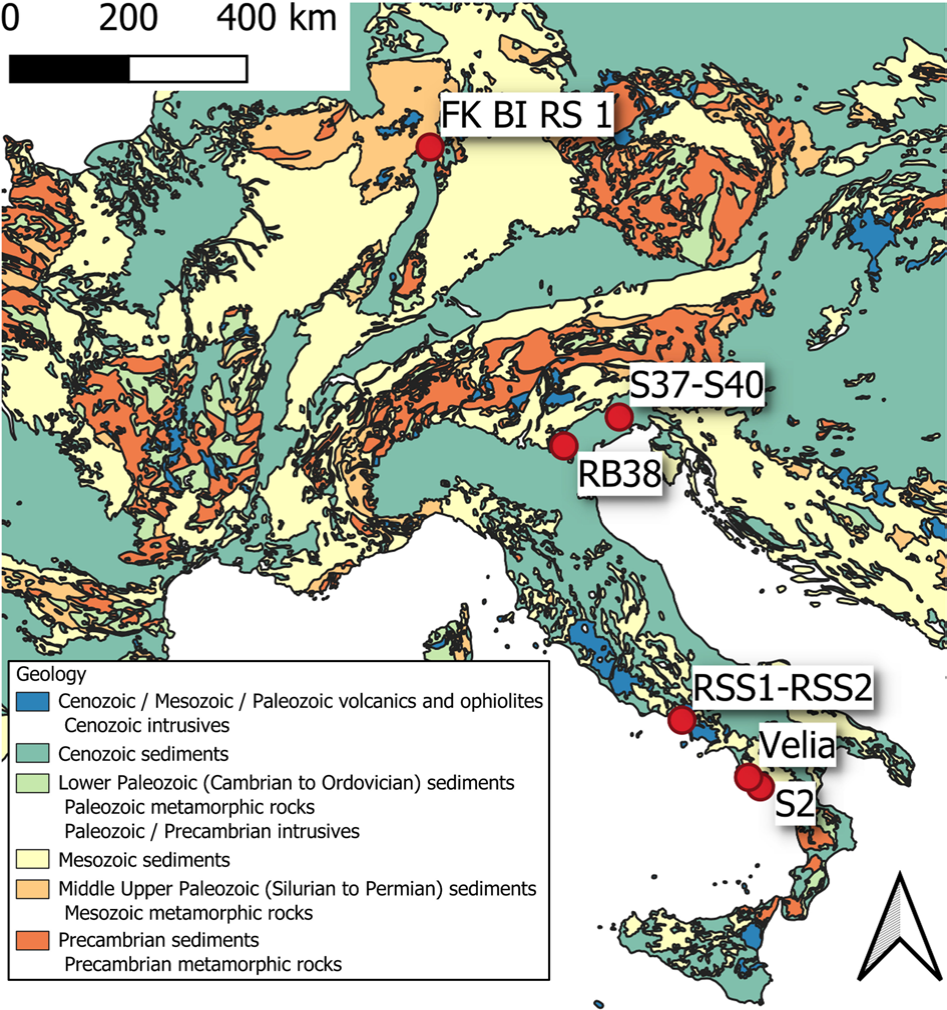
Excavation sites of the skeletal samples considered in this study. The simplified geological map has been drawn using QGIS 3.18 (https://www.qgis.org/) by F.L., based on the geological map from^63^.

In this study, seven archeological bone fragments and one tooth have been analyzed by means of CL immunochemical microscopy imaging and LA–ICP–MS (Table 1 and Fig. 1). Samples come from different ages and sites. Samples from the Middle-Upper Paleolithic were from caves, but from different ecogeographical of Italy with a different climate (i.e., RB38 from the Northern Italy, RSS1 and RSS2 from Southern Italy). Samples RSS1 and RSS2 were obtained from the same bone but they were considered separately, since diagenesis could affect heterogeneously different areas of the same bone.

**Table 1:** Bone and tooth samples analyzed in this study with relevant details.

Sample	Skeletal tissue	Provenance	Burial setting	Age	Taxonomy
FK BI RS 1	Bone	Rhine gravels (Upper Rhine Valley, Germany)	Fluvial sediment	Late Pleistocene	Mammal (*Bison* sp.)
S2	Bone	Grotta della Cala (Salerno, Campania, Italy)	Cave	Middle-Upper Paleolithic	Mammal
S37	Bone	Castello della Motta of Savorgnano (Udine, Friuli-Venezia Giulia, Italy)	Open-air site	Early Middle Ages	Aves
S40	Bone	Castello della Motta of Savorgnano (Udine, Friuli-Venezia Giulia, Italy)	Open-air site	Early Middle Ages	Aves
RB38	Bone	Riparo del Broion (Vicenza, Veneto, Italy)	Cave	Middle Paleolithic	Mammal
RSS1	Bone	Roccia San Sebastiano cave, Mondragone (Caserta, Campania, Italy)	Cave	Middle Paleolithic	Mammal
RSS2	Bone	Roccia San Sebastiano cave, Mondragone (Caserta, Campania, Italy)	Cave	Middle Paleolithic	Mammal
Velia_T440	Tooth	Velia (Salerno, Campania, Italy)	Open-air coastal site	Roman Ages	Mammal (*Homo sapiens*)

### Bone samples

**Figure 2:**
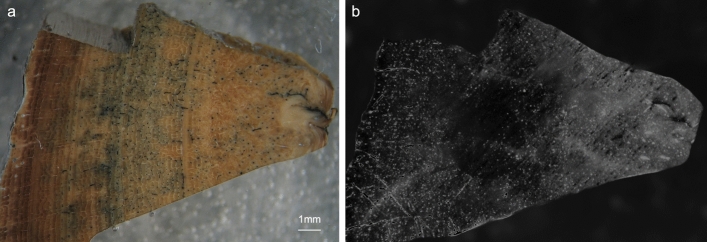
Stereomicroscope (left) and CL (right) images of the investigated sample FK BI RS 1 (1.25 × magnification). Outer surface is on the left and the inner part is on the right. Image 2a has been taken by L.G. following the protocol reported in the “Materials and methods” section.

A Late Pleistocene bison bone (FK BI RS 1), which had been investigated in a previous study^64,65^, was submitted to the analytical protocol proposed. This bone fragment is a cross section of a *Bison* sp. Tibia, exposing the entire cortical thickness (Fig. 2a). The sample was chosen due to its peculiar preservation features having an outer rim (2–3 mm) where, macroscopically, collagen is apparently highly degraded. Yet, the inner cortex portion is better preserved. Such a zonation is also evident from microchemical analyses performed previously on the bone itself^64^. Specifically, the trend of decreasing C/N atomic ratio^66^ from 16.6 at the outer rim to 3.2 towards the center of the bone compacta reflects the strong incorporation of soil-derived humic matter in the outer bone area, also highlighted by the typical dark-brownish coloration^67^. The immunochemical analysis showed indeed the presence of the CL signal mainly localized within the internal part of the bone cortex (Fig. 2b), with a lower content of collagen in the outer part of the specimen.

This agrees with the expected preservation patterns, where collagen is likely to be better preserved in the inner part of bones, namely those areas less affected by alteration processes from the environment and microbial attack^9,36,68^.

Subsequently, line-scan LA–ICP–MS analyses were performed on the sample to quantify its elemental content and cross-monitor the presence of potential elemental gradients linked to the differences in organic matter distribution observed through CL imaging.

**Figure 3:**
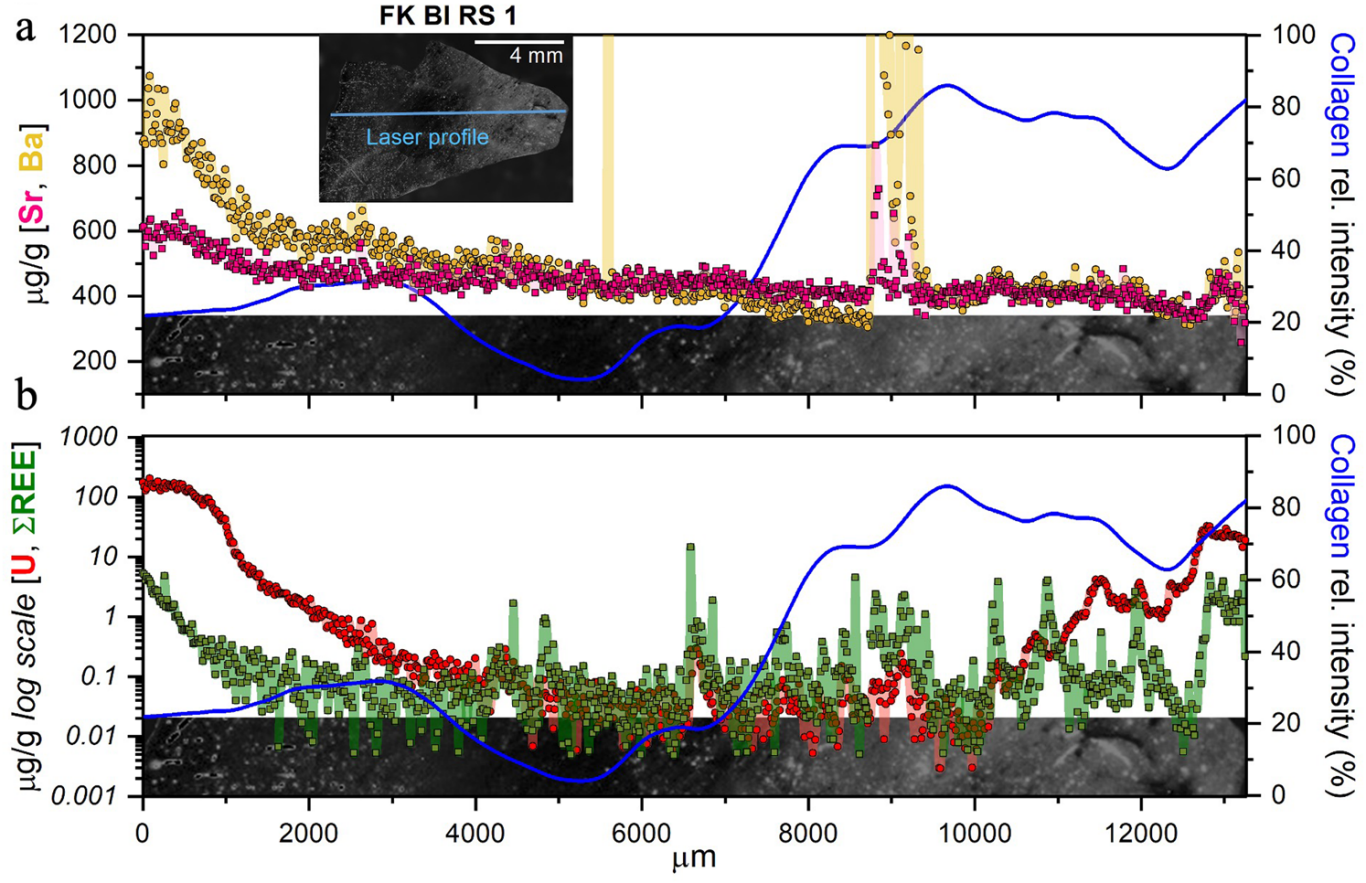
Elemental (Sr, Ba, U and ΣREE) profiles are compared with the CL collagen signal of FK BI RS 1 bone. Outer surface is on the left and the inner part is on the right. Spikes in Sr and Ba profiles at ca. 5600 µm and 9000 µm possibly reflect the presence of post-depositional secondary bone porosity infills by diagenetic minerals or sediment. Note that U and ΣREE are reported in log-scale. Semi-quantitative intensities of the light signal were interpolated from the CL image itself along the LA profile using Icy 2.1.2.0 (icy.bioimageanalysis.org^69^) and reported as relative intensities (scaled to the maximum value). To obtain a representative averaged signal of the area of interest, data from six equidistant lines were collected. Finally, a LOWESS smoothing filter (span 0.1) was applied to the resulting averaged signal.

Our analysis indicated a well-recognizable gradient (Fig. 3a) from the outside towards the inside of the bone fragment with a significant decrease in the mass fraction of Ba and Sr in the innermost better-preserved areas (from 1000 µg/g down to 300 µg/g for Ba and from 600 µg/g down to 375 µg/g for Sr). REE (expressed here as the sum of the REE content, i.e., ΣREE) and U were also measured to better investigate the elemental behavior in well-preserved and altered areas (Fig. 3b). Despite the high variance in mass fraction values along the sample, ΣREE and U clearly decrease several orders of magnitude towards the inner part of the bone (from 200 µg/g down to 0.01 µg/g for U and from 5 µg/g down to 0.01 µg/g for ΣREE). The innermost part of the compacta of FK BI RS 1, close to the medullar cavity, displays a slight enrichment in ΣREE and U. This latter, specifically, shows an increasing mass fraction from ca. 10,000 µm (less than 0.1 µg/g) to the right edge of the sample (up to 20 µg/g). It should be noted that this enrichment is approximately 10 times lower in terms of absolute mass fraction, compared with the outermost bones surface (Fig. 3). Therefore, a different, though less prominent, diagenetic elemental uptake from the medullar cavity—left empty after the decomposition and commonly infilled post-depositional by local soil—occurred.

Several other bones (Table 1) were analyzed by the integrated CL and LA–ICP–MS approach to better elucidate the possible correlation between collagen loss and elemental uptake after burial.

**Figure 4:**
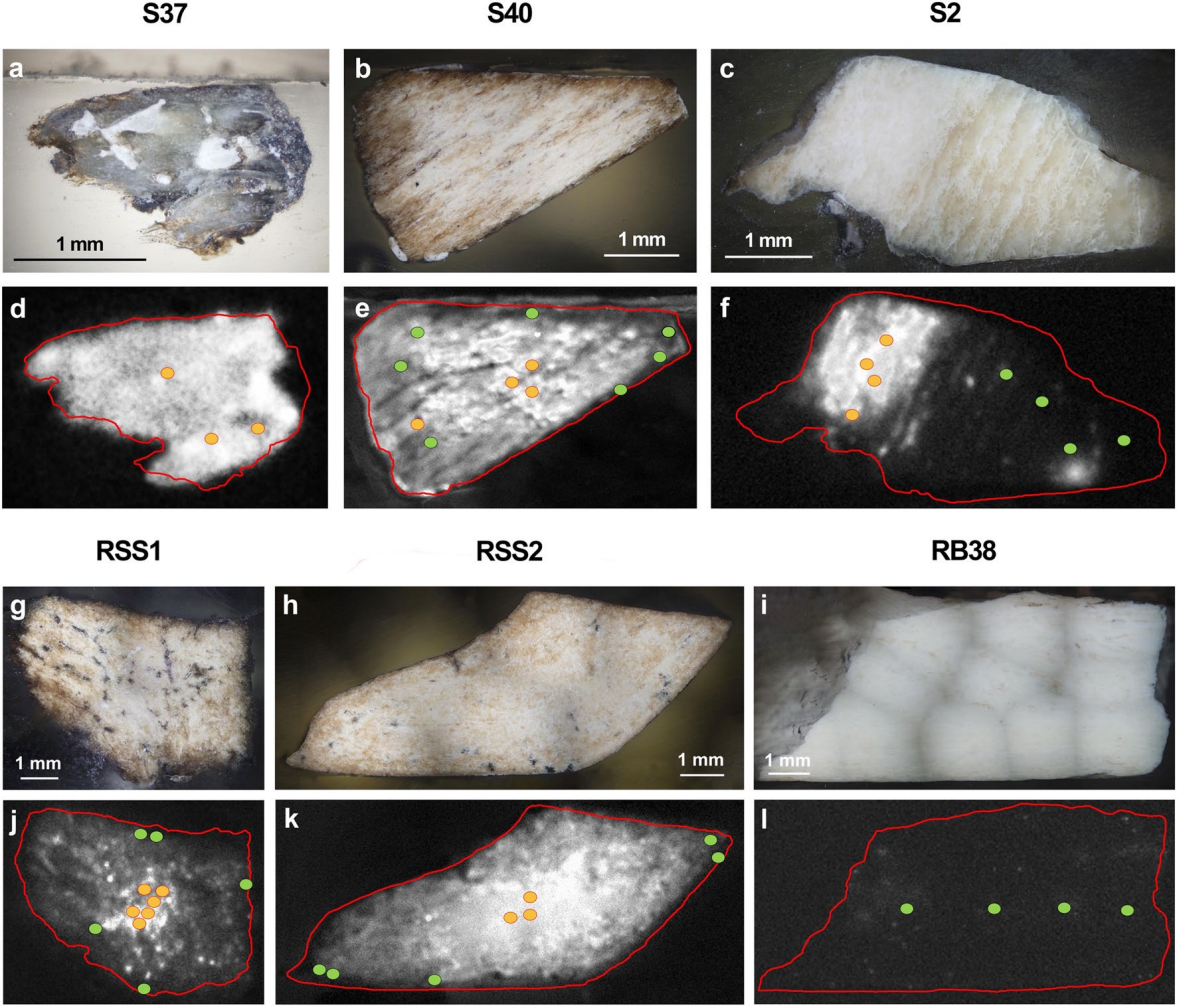
Optical microscope (top) and CL (bottom) images of investigated archeological faunal bone samples with known age and provenance (Table 1): (**a**,**d**) sample S37 (5 × magnification, outer surface is on the top and the inner part is down), (**b**,**e**) sample S40 (1.25 × magnification, outer surface is on the top and the inner part is down), (**c**,**f**) sample S2 (1.25 × magnification, inner surface is on the top and the outer part is down), (**g**,**j**) sample RSS1 (1.25 × magnification, inner surface is on the top and the outer part is down), (**h**,**k**) sample RSS2 (1.25 × magnification, inner surface is on the top and the outer part is down), (**i**,**l**) sample RB38 (1.25 × magnification, inner surface is on the top and the outer part is down). LA–ICP–MS points of analysis are showed in CL images placed in “high” (orange) and “low” (green) collagen content areas.

The use of the CL immunoassay on the selected samples allow to obtain well detectable CL signals for almost all the samples, even though with different spatial distributions (Fig. 4). Sample S37 (Fig. 4a,d) showed an intense CL signal spread all over the sample surface, as expected for a relatively modern bone specimen (Middle Ages). On the other hand, sample S40, coming from the same archeological context of S37, shows a more heterogeneous collagen preservation and distribution (Fig. 4b,e). Sample S2 (Fig. 4c,f) pertains to the Middle-Upper Paleolithic (> 50 ka) and displays two distinct areas with different collagen contents. Specifically, the low-collagen area seems macroscopically characterized by the precipitation of secondary calcium carbonate minerals, as suggested by the yellowish coloration.

Bone fragment RSS1 (Fig. 4g,j) and RSS2 (Fig. 4h,k), which were collected from different areas of the same bone specimen, both exhibited stronger CL signals in the central area of the fragment. Sample RB38 (Fig. 4i,l) did not show any significant CL signal, hence the collagen amount was lower than the limit of detection^54^.

**Figure 5:**
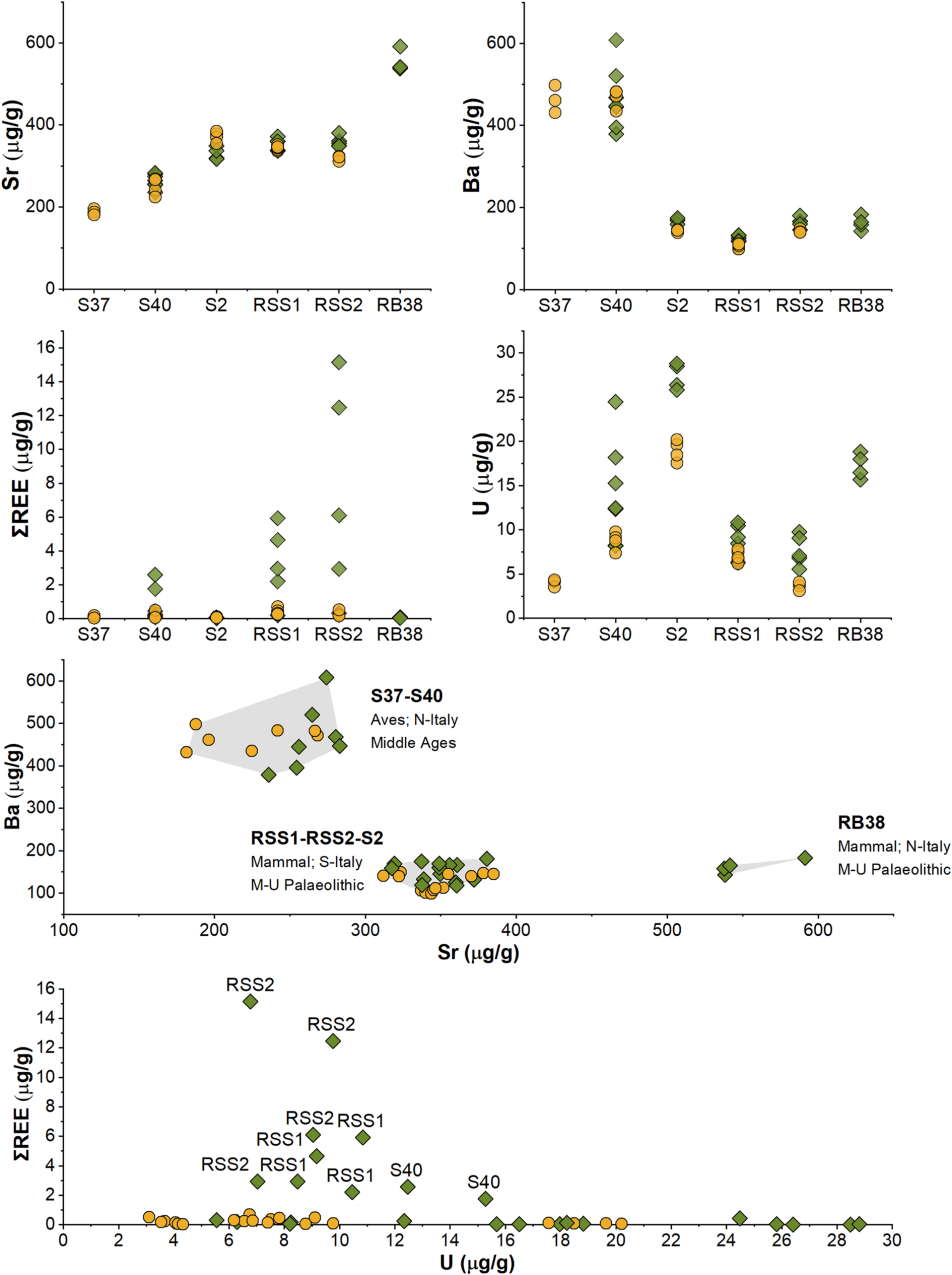
LA–ICP–MS spot analyses (ΣREE, U, Sr, Ba) on “high-” (orange circles) and “low-” (green diamonds) collagen areas of bone samples. These areas were visually selected based on CL images. A biplot of Ba vs. Sr content is reported to highlight inter-sample differences possibly arose *in-vivo* due to inputs such as diet, physiology, or local geology. A biplot of U vs. ΣREE content is also reported to highlight the *t*-test results, showing the differences correlated to high and low collagen content areas in the bone specimens. Error bars (2 SE) are smaller than symbol size. Two-standard errors are calculated based on repeated measures (n = 9) of NIST SRM 1400 bone ash (Sr: 4 µg/g; Ba: 19 µg/g; U: 0.1 µg/g; ΣREE: 0.35 µg/g).

CL immunochemical investigations were integrated with LA–ICP–MS micro-chemical analyses. Ablation spots were selected based on areas with different collagen content based on CL images, aiming to obtain at least three LA spots per area (low vs. high collagen content). This allowed to gather specific information for the areas with differentiated collagen content. For each bone sample, areas characterized by “high” and “low” collagen contents were identified according to the CL results. The identification of areas with high or low collagen content is related to the presence or absence of the CL signal in the investigated area. When the CL signal is not detected (i.e.*,* it is below the detection limit, which is 0.3 ng/spot^54^) the area is classified as low in collagen. Conversely, if the CL signal is easily detectable, the area is classified as having a high collagen content. The identification of the two different area typologies on the basis of the CL signal allowed to guide the selection of areas for the elemental analysis, identifying possible differences in diagenetic pathways among samples (Fig. 5).

Results obtained for these samples are summarized in Table 2. A two-tailed *t*-test suggests that, on average, Sr, ΣREE and U contents are significantly higher in areas with low collagen content (see i.e.*,* Fig. 5). The *t*-test performed for Sr data is likely biased by sample RB38, which shows an enrichment in Sr content (ca. 550 µg/g; see “Discussion”; *p* after RB38 removal is 0.342). Being apparently compromised in terms of organic preservation, for RB38 a comparison with a well-preserved collagen area is lacking. After removing RB38 from the dataset, no significant differences can be observed in Sr content between low and high collagen content areas (two-tailed *t*-test; *p* = 0.342; Fig. 5). In terms of Ba content, low- and high collagen content areas are statistically indistinguishable (two-tailed *t*-test; *p* = 0.932).

**Table 2:** Summary statics of LA-ICPMS spot analyses on bone samples (i.e., S37, S40, S2, RSS1, RSS2 and RB38).

Element	Sr (µg/g)		Ba (µg/g)		ΣREE (µg/g)		U (µg/g)	
Sampling area	Low collagen	High collagen	Low collagen	High collagen	Low collagen	High collagen	Low collagen	High collagen
Mean	358	304	241	245	2.34	0.22	14.4	8.8
St. Dev	96	66	149	168	3.95	0.19	7.4	5.6
Coeff. of variation	0.27	0.22	0.62	0.68	1.69	0.85	0.51	0.64
Median	349	330	167	145	0.25	0.16	12.3	7.1
Max	591	385	608	498	15.16	0.69	28.8	20.2
Min	236	182	118	99	0.02	0.03	5.6	3.1
*t*-test (low vs. high)	*p* = 0.038		*p* = 0.932		*p* = 0.021		*p* = 0.007	

**Figure 6:**
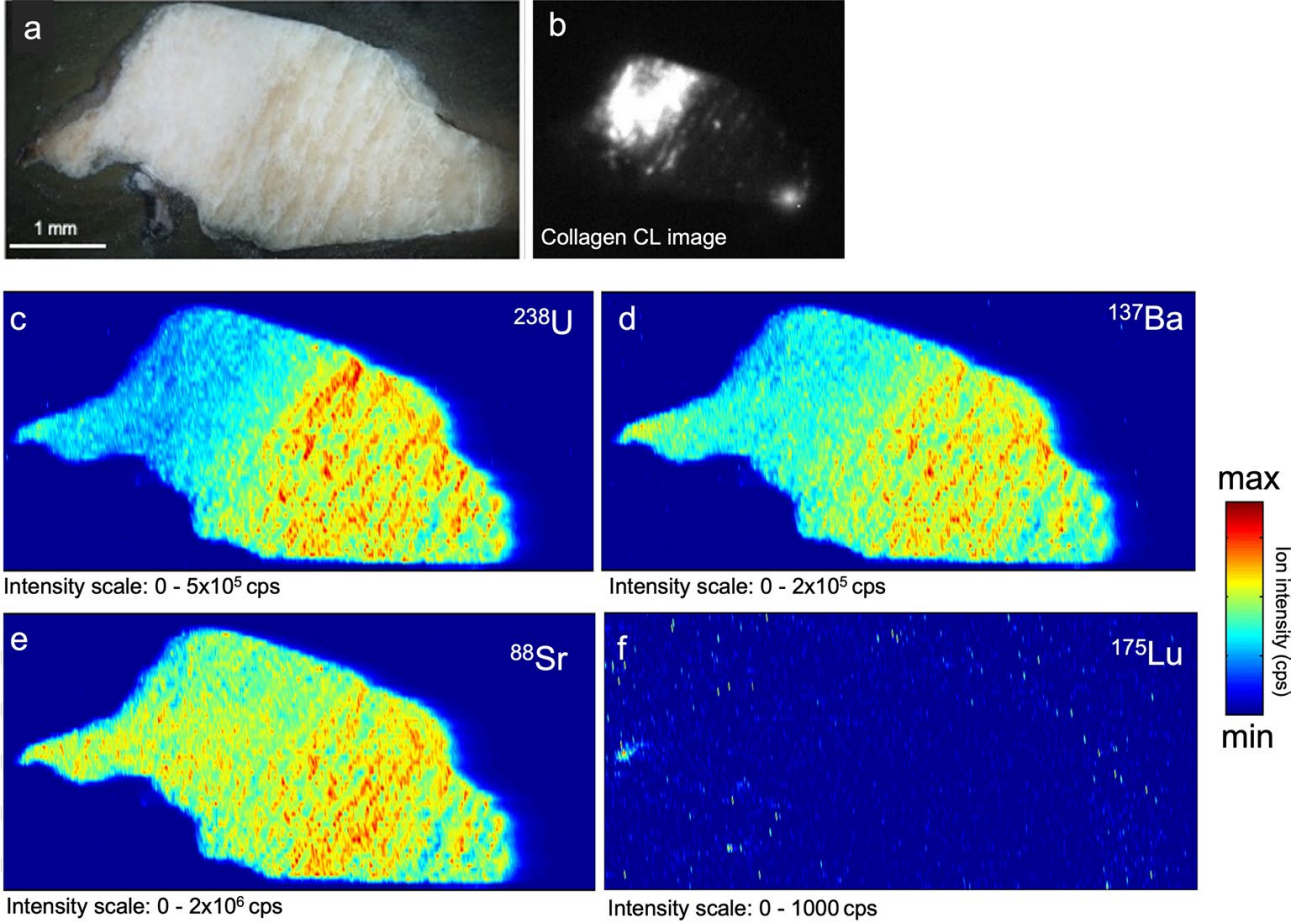
Elemental images of sample S2 obtained by LA-ICPMS. (**a**) Photomicrographs of the sample, (**b**) CL image of the collagen distribution, (**c**) ^238^U, (**d**) ^137^Ba, (**e**) ^88^Sr, (**f**) ^175^Lu (as REE content proxy). Note that the elemental images are not reported normalized to an internal standard but as raw cps (counts per second). Maps were obtained using a MATLAB 2010a script^62^.

Additionally, to further investigate the correlation between elemental and collagen distributions in these bones, sample S2 was subjected to LA–ICP–MS imaging (Fig. 6). It is worth to notice that the normalization method employed for calculating absolute mass fraction in LA spots, i.e., using ^44^Ca as internal reference, might have smoothed potential differences in elemental mass fractions for those samples that have undergone recrystallization events. Ca content was indeed assumed as constant and equal to that of modern bone bioapatite (ω ~ 26.5%).

### Tooth sample

**Figure 7:**
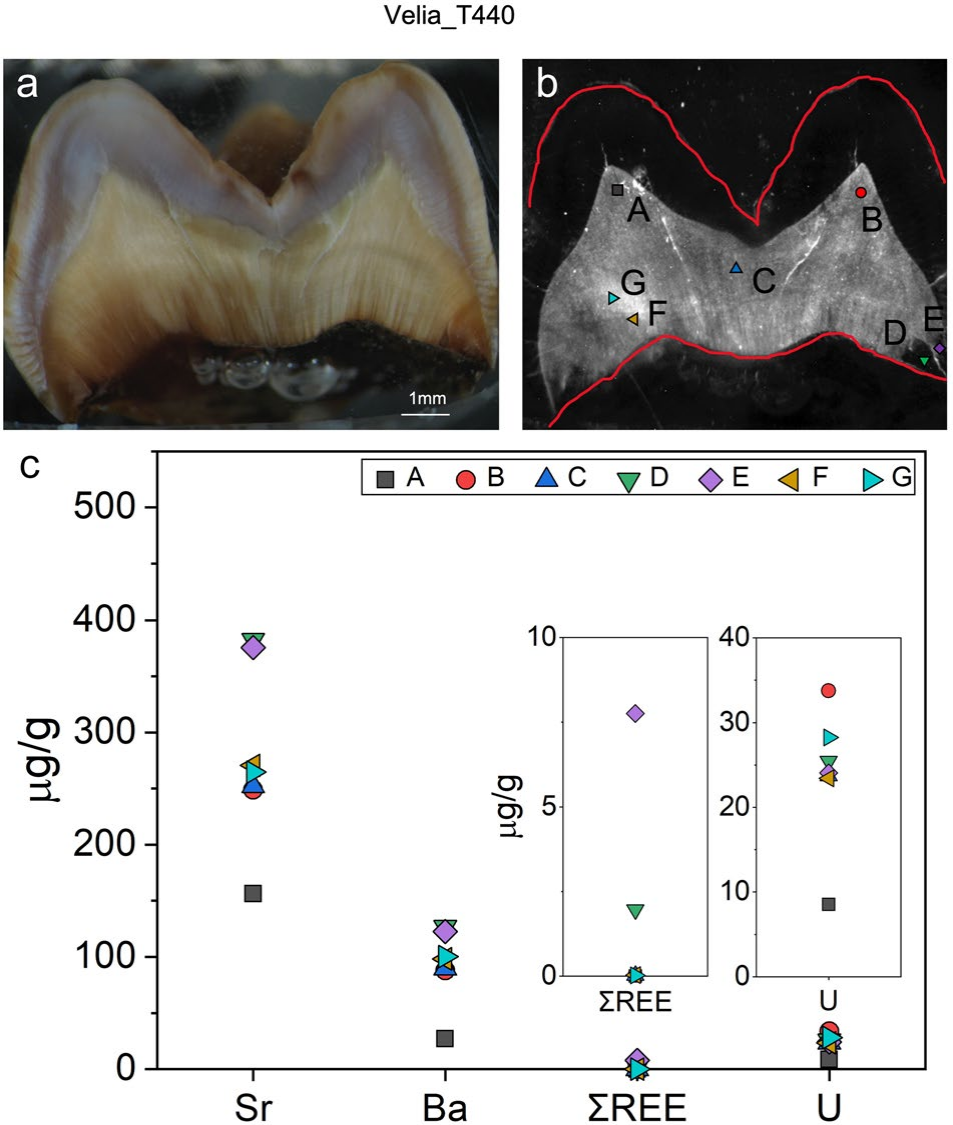
Image of tooth Velia_T440 (1.25 × magnification). (**a**) Optical microscope, (**b**) CL image with points of LA–ICP–MS spot analysis. Bright lines are due to fissures, (**c**) LA–ICP–MS spot analyses (ΣREE, U, Sr, Ba). Error bars (2 SE) are smaller than the symbol size. Two-standard errors are calculated based on repeated measures (n = 9) of NIST SRM 1400 bone ash (Sr: 4 µg/g; Ba: 19 µg/g; U: 0.1 µg/g; ΣREE: 0.35 µg/g).

The integrated method was also applied to one tooth from a Southern Italy site of Roman Age (Velia_T440^70^, Fig. 7a), to evaluate the correlation between collagen and element distribution in different types of skeletal remains. According to the tooth composition, collagen was clearly localized in the dentin as shown by an intense CL signal while, as expected, no signal was detected in the highly mineralized and collagen-free enamel (Fig. 7b).

LA–ICP–MS spot analysis was performed on the dentin tissue (Fig. 7c) according to the intensities of the CL signal, aiming at monitoring the diagenetic markers already observed for the bones. Specifically, data collected from spots D and E, in an area characterized by the absence of CL signal and thus with a likely poor collagen content, present as expected the highest Sr, Ba and ΣREE contents. On the contrary, lower Sr, Ba and ΣREE contents were detected in all other spots, characterized by a relatively high CL signal.

## Discussion

The results of this study clearly indicate different intra- and inter-sample states of preservation, leading to variable collagen and elemental composition even within the same sample. This may be ascribed to the heterogeneity of the degradation phenomena occurring after burial.

Owing to the specificity and the selectivity of the CL immunochemical imaging analysis, it was possible to unequivocally localize collagen^54^ within skeletal samples of variable composition and preservation. It is worth noticing that the anti-collagen type I antibodies have high interspecies cross-reactivity^54^, ensuring the identification of collagen in all the samples belonging to different species. Furthermore, the application of the integrated protocol revealed a strong correlation between organic content and the content of diagenetic marker elements (i.e., REEs and U) in bone and dentin.

Uranium and REE have been already identified as diagenetic markers for bone fossilization, reflecting the surrounding post burial environment^38^. Both, U and REE are usually absent or only occur in very low mass fraction (< 1 µg/g) in fresh bone, but after burial they may be incorporated from groundwater and soil, increasing their mass fractions in specific areas of the buried bone^25,28,37^. Strontium and barium, on the other hand, are biomarkers for dietary reconstruction, *in-vivo* present within bones between tens and hundreds of µg/g (even less for Ba). However, their endogenous signature can be overprinted by post-depositional exogenous processes^16,24^. This work revealed for the first time that diagenetic elemental markers for bioapatite could be applied for collagen preservation studies, due to their observed correlation with CL collagen signals. In our data, FK BI RS 1 showed high Sr and Ba values in the outer rim, coupled to U and REE enrichment, due to incorporation of elements from the surrounding environment within the bioapatite structure, possibly linked to a simple diffusion uptake model, as previously explained also by Ullman and colleagues^24,26^. However, other bone samples showed remarkably variable Sr and Ba contents and lack of correlation with the degree of collagen preservation, highlighting a possible different diagenetic pathway. Indeed, differences in burial setting and/or *postmortem* element uptake processes could influence the differential diagenetic alteration of Sr and Ba versus REE and U^71^. As expected, in LA–ICP–MS data a relatively high variability (see the coefficients of variation in Table 2, calculated as the std. deviation divided by the mean) was observed, as samples come from different taphonomic and temporal contexts. Moreover, as expected, diagenetic markers (i.e.*,* REEs and U) show higher variability than Sr and Ba. The LA–ICP–MS mapping results on S2 emphasized what observed by spot analyses. Interestingly, U was preferentially distributed in those areas lacking collagen preservation, mimicking the macroscopically visible vein-like diagenetic infilling further corroborating the negative correlation between collagen and trace element diagenetic markers. Similarly, Ba seems more concentrated in the diagenetically altered portions. REEs content (here lutetium distribution) in S2 is too low to be meaningfully interpreted. Strontium seems more homogeneously distributed in the sample, with some higher contents within the secondary veins. Moreover, in CL results the variability of collagen content was evident among samples from sites of similar ages (RB38, RSS1, RSS2) and also within the same specimen (RSS1, RSS2), suggesting that the environmental conditions and the post-burial histories of the bone samples may lead to very different conservation status. For instance, the absence of signal in RB38 agrees with the whitish coloration displayed, possibly indicating an exposure to high temperatures^72^ that caused the degradation of organic matter. The different taphonomic contexts need also to be considered. Indeed, the local environmental setting (i.e., pH, temperature, humidity, soil composition) and the taxonomy (i.e., bone structure) can lead to different diagenetic pathways and histories^16,24,28,35^. At the same time, the variability of the numerous in-vivo factors (i.e., diet, mobility, physiology), hamper the decoupling of the unaltered bone elemental signals and the diagenetic overprints. In general, our data indicate a negative correlation between the preservation of collagen (and, more generally, organic fraction) and the elemental uptake during diagenesis. A similar result was obtained in previous research^26^, in which a possible correlation between REE and collagen was stated. However, this hypothesis has not been based on the direct and spatially resolved correlation between elemental and immunochemical data, for a specific bone area. On the contrary, the analytical approach presented here initially involved a sensitive spatial localization of collagen in different areas of the same sample, based on which the investigation of chemical diagenetic markers was conducted, thus obtaining effective information on the correlation of data with a high spatial resolution. As highlighted, specific diagenetic histories need to be evaluated from case to case. For example, the low REE content of sample RB38, associated with an almost null CL signal across the whole specimen, indicates that the local diagenetic end-member is characterized by a depleted REE signature. Yet, the elevated U and Sr contents still suggest that these samples underwent strong post-depositional modifications.

Because of the growing interest for teeth as biological archives of information, the combined organic and inorganic preservation of a tooth sample (Velia_T440) was investigated in order to better clarify dentin resistance to diagenetic alterations. Like bones, dentin contains high amounts of proteinaceous materials (ca. ω = 20%) and is prone to post-burial alterations, while enamel is a hard tissue composed by a small organic fraction (ω < 5%), whose major components are amelogenins^73^. The CL signal appears quite homogeneous, except for small areas macroscopically characterized by a darkish coloration. As expected, no CL response is visible within the tooth enamel. Results confirm the negative correlation between CL collagen signal and the relative enrichment of tracers for diagenetic alteration such as REEs. However, a site-specific diagenetic end-member might influence the preservation of in-vivo elemental signals, as in the case of Velia_T440.

It is worth to notice that U showed an unexpected trend in Velia_T440 compared to bones. Indeed, the highest U content is displayed in spot B, which is localized in a high-collagen area with low ΣREE, Sr, and Ba contents. For this tooth, ΣREE seems to better predict collagen degradation rather than U. Specifically, a remarkably high U content (up to ca. 35 µg/g) was observed in the dentin, despite the relatively recent age of the sample. This may suggest that local groundwaters were rich in U, maybe due to geologically-related (i.e., volcanic) sources in the Campanian area^74^. Despite the seemingly poor elemental preservation of Velia_T440, collagen is still present and quite homogeneously distributed. Such evidence may indicate that, in this case, the diagenetic pathway was characterized by surface adsorption and/or complexations of ions with organic molecules (such as humic acids), with little collagen hydrolysis and limited reprecipitation of secondary mineral phases^75^.

The results have shown that the innovative integration between CL microscope immunochemical detection and LA–ICP–MS analysis allows a significant improvement of the information achieved regarding the degree of diagenetic alteration in bones and teeth, thus clarifying and demonstrating for the first time the mutual relation between variations in element mass fraction and collagen preservation.

To sum up, the diagenetic proxies considered in this study support the idea that post-burial collagen degradation is linked with the alteration of the bone in-vivo element composition. In particular, the diagenetic markers REE and U seem to show a stronger negative correlation with the presence of collagen than Sr and Ba, which cannot always be associated with diagenetic variations. However, case-specific post-burial pathways might lead to different resulting elemental contents and collagen preservations. Our work shows that the combination of in-situ CL immunochemical imaging, for the first time applied on archeological samples, and elemental analyses may help to unravel bone diagenesis history and understand the link between the primary organic content and the alteration of the mineral phase. The combination of CL imaging and LA–ICP–MS indicated that REE and U contents of bone or dentin may be used as diagnostic chemical markers for collagen preservation. Thus, based on the results of this study, LA–ICP–MS elemental data can be used to better constrain least altered bone or dentin areas with higher collagen contents, according to the mass fraction of these diagenetic markers.

Exploiting the potentialities of CL imaging, future research studies will be focused on the combination of CL immunochemical analysis with NIR hyperspectral imaging to compare and integrate the information obtained to properly select well preserved area in bones and dentin to be submitted to C- and N-isotope analysis, proteomics or DNA investigations.

## Materials and methods

### Sample preparation

Small fragments were obtained from each bone sample, while the tooth has been cut in half. The fragments were then embedded in Implex® polyester resin (Remet S.A.S., Bologna, Italy) and polished with abrasive paper Micromesh™ from 120 to 12,000 grit to obtain a smooth surface^76^. Visible images of the smaller cross-sections were acquired with an Olympus BX51M optical microscope (Olympus Corporation, Tokyo, Japan) connected to an Olympus DP70 digital camera and processed through Autopano Giga 4.2 and Adobe Photoshop 2020. Visible images of the sample with a larger dimension (bison and tooth) were acquired with a stereo-microscope Leica® MZ6 connected to a Canon® power shot 550 digital camera.

### Assay procedure

Samples were first incubated at room temperature under stirring for 1 h with a phosphate buffered saline (PBS)/milk5 blocking solution (PBS containing c = 5% (g/L) non-fat dried milk (Sigma-Aldrich)). PBS for preparation of blocking solution and antibody solutions contained 10 mmol/L phosphate buffer and 137 mmol/L NaCl, with pH adjusted to 8.1. After washing for 5 min with PBS under stirring, they were incubated overnight at 4 °C with the rabbit anti-type I collagen polyclonal antibody (primary antibody, 0.90 mg/mL stock concentration, ab34710, AbCam, Cambridge, UK) diluted 1000 times in PBS/milk1 (PBS containing c = 1% (g/L) non-fat dried milk). Afterwards, the samples were washed (4 ×) with PBS under stirring and incubated for 4 h at 4 °C with the polyclonal horseradish peroxidase (HRP)-labeled anti-rabbit IgG antibody produced in goat (secondary antibody, 0.68 mg/mL stock concentration, 12-348, Sigma-Aldrich Co., St. Louis, MO) diluted 500 times in PBS/milk1. Negative control tests for assessing the specificity were instead performed by using the polyclonal anti-chicken ovalbumin rabbit antibody (1.0 mg/mL stock concentration, ab181688, AbCam, Cambridge, UK) diluted 1000 times in PBS/milk1 as primary antibody^55^. After incubation with the immunoreagents, samples were washed again (4 ×) with PBS, then 50–100 µL of the SuperSignal ELISA Femto CL substrate for HRP (ThermoFisher Scientific, Inc., Rockford, IL) was added to completely cover the cross-section. Finally, a sequence of CL images (image integration time 30 s) was acquired using Olympus BX51M optical microscope (Olympus Corporation, Tokio, Japan) connected to a cooled ultrasensitive monochromatic Retiga Lumo™ CCD camera (Teledyne, Photometrics, Tucson, AZ). The microscope was enclosed in a homemade dark box to exclude any interference from ambient light. Live images of the samples were also acquired to assess the localization of the CL signals. To directly compare elemental and CL data of samples BI RS 1, semi-quantitative intensities of the light signal were interpolated from the CL image along the laser ablation profile area using Icy 2.1.2.0 (icy.bioimageanalysis.org) and reported as relative intensities (i.e., scaled to the maximum value). To obtain a representative averaged signal of the area of interest, data from six equidistant lines were collected. Finally, a LOWESS smoothing filter (span 0.1) was applied to the resulting averaged signal using Origin 2020.

### Laser ablation ICP–MS analyses

The LA–ICP–MS elemental analysis was conducted using a 213-nm laser ablation system (New Wave Research Inc., Fremont, CA) coupled to an X-Series^II^ quadrupole ICP–MS (Thermo Scientific) housed at the Centro Interdipartimentale Grandi Strumenti of the University of Modena and Reggio Emilia. A circular spot size of 80 µm, an energy density of about 5 J/cm^2^ and a repetition rate of 10 Hz were employed. Helium (0.6 L/min) was used as carrier gas. To avoid external contaminations, sample surface was carefully pre-ablated before each analysis. The following masses were collected to possibly detect post-depositional diagenetic modifications: ^44^Ca, ^88^Sr, ^137^Ba, ^139^La, ^140^Ce, ^141^Pr, ^146^Nd, ^147^Sm, ^153^Eu, ^157^Gd, ^159^ Tb, ^163^Dy, ^165^Ho, ^166^Er, ^169^Tm, ^172^Yb, ^175^Lu and ^238^U. Backgrounds were corrected by subtracting the on-peak baseline signals collected during 45 s acquisition with laser off. Data were then internally calibrated using ^44^Ca (ω = 26.9% for dentin and ω = 26.5% for bone) and externally through NIST SRM612 (trace elements in glass), following^77^. Relative standard deviations measured on NIST SRM 612 are better than 10%. Results of NIST SRM 1400 (bone ash pressed pellets, quality control) are reported in Table 3 and compared with reference values from the GeoReM database^78^. Limits of detection were calculated as three standard deviations of the blank signal, following^77^. A two-tailed *T*-test was performed on elemental data of low vs. high collagen areas, using a MATLAB script. Only data above the LOD were considered. Laser ablation imaging was performed with the same instrument, following the protocol described in Sforna and Lugli^62^. Specifically, the bone sample was mapped using unspaced line scans with a spot size of 50 µm, covering the whole sample surface. Maps were then built in the MATLAB environment, using MapIT! (see^62^).

**Table 3:** Compositional data of the quality control reference material (NIST SRM 1400, bone ash).

Element	Average mass fraction (µg/g)	C.I. 95% (µg/g)	Reference values in µg/g (from GeoReM)	LOD (µg/g)
Sr	241	237–245	210–274 (compiled: 249)	0.1
Ba	243	223–262	240–308 (compiled: 240)	0.01
La	0.407	0.223–0.592	0.01–0.46 (compiled: 0.386)	0.005
Ce	0.710	0.446–0.974	0.009–0.94 (compiled: 0.821)	0.001
Pr	0.077	0.046–0.108	0.008–0.094 (compiled: 0.086)	0.001
Nd	0.293	0.170–0.415	0.009–0.342 (compiled: 0.316)	0.003
Sm	0.059	0.028–0.090	0.008–0.0664 (compiled: 0.0595)	0.002
Eu	0.013	0.004–0.021	0.0136	0.001
Gd	0.050	0.034–0.066	0.006–0.071 (compiled: 0.064)	0.004
Tb	0.006	0.004–0.008	0.00885–0.01 (compiled: 0.0096)	0.0006
Dy	0.058	0.040–0.076	0.009–0.0524 (compiled: 0.0479)	0.004
Ho	0.010	0.006–0.014	0.009–0.012	0.001
Er	0.023	0.018–0.028	0.008–0.0276 (compiled: 0.0254)	0.003
Tm	0.005	0.002–0.007	0.003–0.009 (compiled: 0.0034)	0.002
Yb	0.022	0.014–0.029	0.006–0.0237 (compiled: 0.0183)	0.005
Lu	0.005	0.001–0.010	0.0034–0.009	0.002
U	0.060	0.038–0.082	0.062–0.076 (compiled: 0.066)	0.0007

Average and confidence interval (C.I.) are calculated based on 9 spot analyses (i.e., replicas). Limit of detections (LOD) are reported as 3σ of the blank.

## Data Availability

The datasets generated during and/or analysed during the current study are available from the corresponding author on reasonable request.
